# Management of radioiodine ablation therapy in haemodialysis patients with thyroid cancer: a case series of two patients

**DOI:** 10.1186/s12882-025-04348-0

**Published:** 2025-07-28

**Authors:** Raymond Lin, Alessandra L. Malaroda, William J. Ryder, Veronica C. K. Wong, Nikki L. Wong

**Affiliations:** 1 Nepean Kidney Research Centre, Nepean Hospital, Sydney, New South Wales Australia; 2 Department of Nuclear Medicine and PET, Nepean Hospital, Sydney, New South Wales Australia; 3 Sydney Medical School, University of Sydney, Sydney, New South Wales Australia; 4 School of Physics and Centre for Medical Radiation Physics, University of Wollongong, Wollongong, New South Wales Australia

**Keywords:** Haemodialysis, Radioiodine therapy, Thyroid cancer, Nuclear medicine, Case series

## Abstract

**Background:**

Radioiodine (^131^I) therapy in treatment of thyroid cancer, has a biological clearance that is significantly reduced in end-stage kidney disease (ESKD), leading to increased radiation exposure and potential myelotoxicity. For ESKD patients on haemodialysis (HD), there is no standardized approach to ^131^I administration and subsequent HD schedule.

**Case presentation:**

Two patients with ESKD on HD were treated with ^131^I therapy for thyroid cancer. Rationale for treatment and local ^131^I treatment protocol modifications are discussed. Modifications were made to existing infrastructure and additional patient and staff safety precautions were undertaken, including serum radioactivity measurements to monitor for myelotoxicity.

**Outcomes:**

HD at 24-,72- and 144-hours post-^131^I results in a retained radiation activity profile comparable to patients with normal renal function. Radiation dose to bone marrow throughout treatment was assessed at < 0.3 Gy for both patients, within safe limits. The highest contribution of radiation dose to bone marrow (60% and 47% for patient 1 and patient 2 respectively) was due to the radioactivity retained in blood before the first HD session. Cumulative radiation exposure to dialysis staff was well within local safety constraints (300μSv per year) at 7μSv and 23μSv for patient 1 and 2 respectively. At 24 months post-therapy, thyroglobulin levels remained undetectable for both patients.

**Conclusions:**

^131^I therapy can be safely administered in patients with ESKD on HD with low-risk thyroid cancer through modifications to existing infrastructure and protocols. Serum radioactivity measurements is a simple and minimally invasive method to assess bone marrow safety during treatment. Ongoing pooling of experiences is needed to inform a standardized protocol for therapy in this population.

## Background

Ablative radioiodine (^131^I) therapy with ^131^I-sodium iodide following thyroidectomy is often considered in patients with differentiated thyroid cancer (DTC) following total thyroidectomy, and may be a requirement for patients being considered for renal transplantation. As radioactive ^131^I is cleared primarily via the kidneys, there may be prolonged blood retention of ^131^I in patients with end-stage kidney disease (ESKD) requiring haemodialysis (HD), resulting in increased risk of myelotoxicity [1]. In this population, timing of HD sessions is critical since the extraction efficiency of radioactive ^131^I by HD is greater than that of normal kidney function and, if scheduled too early, may compromise treatment efficacy. In the days immediately after ^131^I administration, patients may also pose a radiation risk to nursing staff during HD, being themselves a source of γ-radiation from radioactivity still retained in blood. Radioiodine treatment in HD patients thus presents logistical and clinical challenges for patients and clinical staff.

There is limited published guidance in the treatment procedures for radioablation of thyroid remnants in ESKD patients [2, 3] and literature is limited to case reports and case series featuring a range of protocols and approaches [4]. The paucity of literature in treating these patients contributes to the lack of consensus in the optimal treatment schedule to maximize treatment efficacy while limiting risks of myelotoxicity [5]. With the objective of contributing our experience to the existing literature and the goal that pooled data from studies may build toward the development of a standardized treatment protocol for this patient population, we report a case series of two HD patients who underwent ^131^I therapy in a tertiary centre in Sydney, Australia. The modified protocol for ^131^I therapy in two low to intermediate risk HD patients is described, reporting on staff and patient safety measures and the outcomes of treatment.

## Case presentation

Two HD patients with DTC presents for post-thyroidectomy adjuvant ^131^I therapy. Baseline characteristics, tumour characteristics, cancer staging and HD prescriptions for both patients are shown in Table 1. Patient 1 was anephric due to bilateral nephrectomy. Patient 2 was morbidly obese (body mass index 50) and had a colostomy in situ. Patient 1 was taught to and successfully self-cannulated for all HD sessions ahead of treatment.

**Table 1:** Baseline characteristics, cancer characteristics and haemodialysis prescriptions of two haemodialysis patients undergoing radioiodine therapy

	Patient 1	Patient 2
Age (*years*)	41	60
Gender	Male	Male
Prescribed activity (GBq)	1.0	1.0
rhTSH/thyroxine withdrawal	rhTSH	rhTSH
Dry weight (*Kg*)	98	163
Estimated blood volume (*L*)	5.8	7.1
Urine output (*mL/day*)	0	630
24 hr urine creatinine clearance (*mL/min*)	0	6
Dialysis duration (*hours*)	5	4.5
Dialysis membrane size (*m*^*2*^)	2.5 (Solacea 21 H)	2.1 (FX CorDiax 120)
Blood flow speed (*mL/min*)	300	300
Vascular access type	Radiocephalic AVF	Radiocephalic AVF
Thyroid cancer type	Papillary	Papillary
Primary tumour size	12 × 8 × 6 mm	26 × 25 × 18 mm
Lymph node involvement	Yes (1 of 5) Deposit size 2 mm	Undefined
Stage (*AJCC 8*^*th*^* Edition TNM*)	I (T1b, N1, M0)	II (pT2, NX, M0)
Notable considerations	Anephric Transplant candidate	Morbid obesity Stoma
MDT input	Requested by transplant team prior to listing for transplant, considered low to intermediate risk of recurrence	Tumour within 1 mm of anterior and posterior margin, positive for BRAF, patient preference for active treatment, considered low to intermediate risk of recurrence

(AVF = arteriovenous fistula, AJCC = American Joint Committee on Cancer, MDT = multidisciplinary team)

The comparator population was eight consecutive patients without renal impairment that underwent ^131^I therapy for thyroid cancer in the four months preceding, at the same centre. The comparator population had a median age of 55.5 years with seven females and one male. Six patients had prescribed activity of 4.0GBq and two patients had prescribed activity of 2.0GBq. Clearance of ^131^I from these patients were used for a qualitative visual comparison of clearance characteristics against the two HD patients.

### Rationale for radioiodine therapy

The decision for therapy was reached through multidisciplinary team discussion with endocrinologists, surgeons and nuclear medicine physicians for both patients. Both patients were assessed as low to intermediate risk for recurrence of thyroid carcinoma based on the American Thyroid Association (ATA) risk stratification system, with the aim of ^131^I therapy being remnant thyroid ablation [6].

For patient 1, the decision for treatment was made due to lymph node positive disease and to enable kidney transplant wait-listing due to eligibility criterion set by their transplantation unit. Whole-body imaging during ^131^I therapy additionally provided an opportunity to assess for metastatic disease in a young kidney transplant candidate. Patient 2 was not a kidney transplant candidate and decision for treatment was made on the histological risk factors of close surgical margins (1 mm), BRAF positivity and lack of lymph node information, with the patient preferencing therapy in this context.

### Pre-treatment preparation

Several multidisciplinary consultations were conducted with the nuclear medicine physician, medical physicist, nephrologist and clinical nurse consultant to formulate patient-specific treatment plans, with the aim of establishing a local protocol for treatment in this patient population.

Considerations included ensuring an appropriate location with required shielding to prevent radiation exposure to the public while maintaining HD requirements of patients. While home HD was considered, it was recognized that in most cases, it would not be possible give outpatient ^131^I and ensure compliance with New South Wales (NSW) radiation safety legislation that are based on international guidance [7].

Costs and impact on general hospital functioning were other considerations, with a not insignificant cost in making modifications to the existing radioisotope treatment room and additional dialysis staff rostering for one-to-one care of patient during HD in isolation. Existing treatment protocols were modified to ensure written guidance on the safe management of these patients in isolation as well as safety of staff.

#### Room preparation

**Fig. 1:**
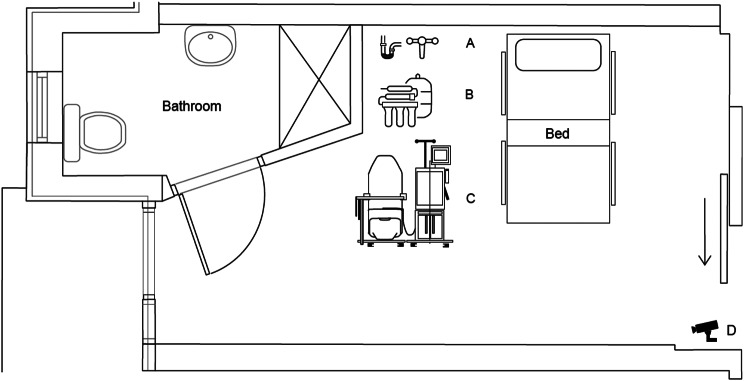
Floor plan of lead-lined treatment room. **A** – water supply and drainage unit. **B** – portable reverse osmosis machine. **C** – haemodialysis machine and chair. **D** – CCTV monitoring

Specific modifications were made to our hospital’s existing lead-lined room for radioiodine treatment. Plumbing to create a water supply and safe drainage for HD was installed under the supervision of the Radiation Safety Officer. A portable reverse osmosis machine (Baxter/Gambro WRO 300 H) for water purification and HD machine (Fresenius Medical Care 5008S) was sourced. An important consideration is the isolation period required for both units after treatment completion; contaminated equipment is typically stored for ten half-lives (approx. three months for ^131^I). There was existing closed-circuit television (CCTV) monitoring in the room. The treatment room set-up is shown in Fig. 1.

#### Staff safety and education

The following additional precautions were implemented for staff safety:

1. Radiation safety education sessions for all staff involved were conducted by the hospital Medical Imaging Physics Service.
2. Disposable personal protective equipment (PPE) was worn at all times within the room (face-shield, mask, gown, double-layered gloves and shoe covers) to protect against splash contamination.
3. HD nursing staff were given electronic personal dosimeters worn underneath PPE to monitor cumulative radiation dose during each HD session.
4. A physicist was available during all HD sessions to monitor staff radiation exposure and intervene in case of radioactive spill/contamination.
5. An area immediately in front of the lead door to the radioiodine room was covered with absorbent paper (Whatman® Benchkote) to allow for assessment of contamination of staff when exiting the room.
6. To minimize staff contact with the patients, patients were assessed for eligibility for self-cannulation and pre-trained if they were suitable.

#### Handling of HD consumables

All HD consumables in the ^131^I treatment room were prepared and stored in the room before admission of the patient, limiting the time spent by nursing staff in the room during the HD sessions. Following HD, all consumables were disposed in a sharps bin labelled as mixed radioactive-biological waste. The waste was stored for ten half-lives (three months) and disposed as biological waste.

#### Handling of pathology specimens

For blood samples (serum biochemistry, full blood counts, coagulation studies) taken during HD, additional provisions were made for handling, isolation, and appropriate disposal in consultation with pathology laboratory staff. Radioactive samples were labelled and safely transported to the laboratory and were processed separately to avoid impacting the results of non-radioactive samples. Once processed, blood samples were collected by Nuclear Medicine staff and disposed appropriately.

#### Other waste

Any biological waste, including colostomy bags, were collected in bins labelled as mixed radioactive-biological waste and safely stored for ten half-lives of ^131^I (three months). At the end of the storage period, radiation level in the waste was re-assessed and, if decayed below NSW regulatory limits, disposed as biological waste. Non-biological waste was stored in double-layered bags and disposed of as general waste.

### Treatment protocol

#### Pre-treatment

A 24-hour urine creatinine clearance was measured one week before treatment to determine residual renal function. Pre-treatment with thyrotropin alfa (Sanofi Genzyme – Thyrogen®) to stimulate remaining thyroid cells was given 48-hours before treatment. Thyrogen® dose was reduced to a single intramuscular dose of 0.9 mg in patient 2 (instead of standard two doses), accounting for significantly slower elimination in patients with ESKD [8]. All comparator patients were given the standard two doses of thyrotropin alfa pre-treatment.

Patients’ usual medications were continued but phosphate binders were withheld for the second patient due to potential binding effect observed in patient 1.

#### Haemodialysis planning

The degree of clearance of ^131^I by HD was taken into account, particularly noting that HD would be necessary to facilitate the majority of clearance of ^131^I. Routine HD was performed 24-hours before ^131^I therapy and on Days 1, 3 and 6, corresponding to 24-, 72- and 144-hours after ^131^I administration. The rationale for this approach is discussed later.

#### Treatment

Administered activity of 1GBq for both patients was decided based on the ATA risk stratification for low to intermediate risk for recurrence of malignancy.

^131^I-sodium iodide was administered orally on Day 0. A nuclear medicine whole body scan was acquired at 4- and 24-hours post-administration and dose-rates at 1 m were measured at 1- and 4-hours. Patients with residual renal function were instructed not to void urine before the dose-rate measurement at 4-hours to allow determination of the calibration factor between administered activity and dose-rate measurement.

Dose rates at 1 m were taken on Days 1, 2, 3 and 6. On HD days (1,3 and 6), the dose-rates were taken both pre- and post-HD. Patients were assessed for potential discharge from Day 3 by the hospital Medical Imaging Physics Service in compliance with state radiation safety legislation (*Radiation Control Act 1990 (NSW), Radiation Control Regulation 2013 (NSW)*). Dose-rate measurements were not scheduled on Days 4 and 5 (Saturday and Sunday) due to resource constraints. If the patient was not discharged on Day 3, they remained in hospital until Day 6.

Blood samples for bone marrow dosimetry were collected on HD days (Days 1, 3 and 6) pre- and post-HD. A standard of care nuclear medicine whole body scan was acquired on Day 3.

**Fig. 2:**
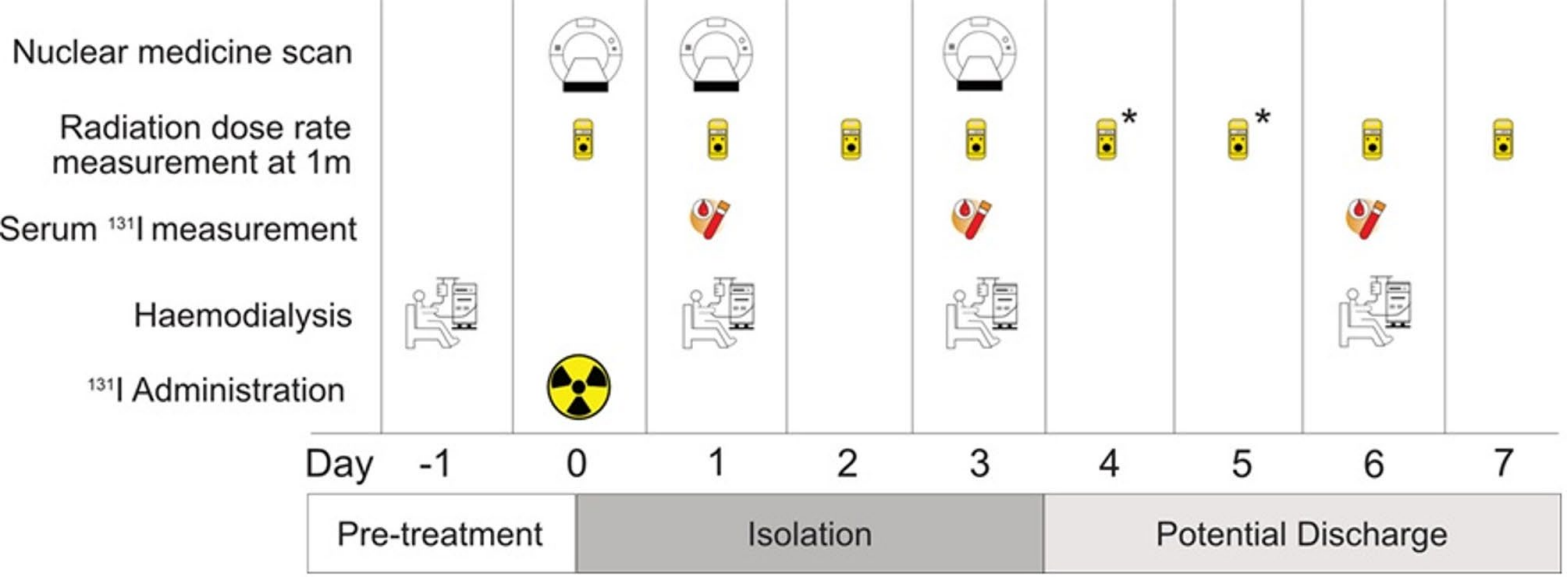
Radioiodine treatment timeline. Haemodialysis sessions were carried out on Day − 1, 1, 3 and 6. Patients were assessed for potential discharge from Day 3. (*) Dose-rate measurements were not taken on Days 4 and 5 due to resource constraints but should be taken if resources are available to facilitate earlier hospital discharge

The treatment timeline over seven days is shown in Fig. 2. The treatment protocol was granted approval by the Nepean Blue Mountains Local Health District Human Research Ethics Committee (HREC) prior to commencement.

#### Assessment of radiation dose to bone marrow

Approval from the Nepean Blue Mountains Local Health District (HREC) was sought for the collection of data for the assessment of radiation dose to bone marrow. Following the European Association of Nuclear Medicine (EANM) guidance [9], radiation dose to blood was assessed as an upper limit to the radiation dose to bone marrow.

## Outcomes

### Pre-treatment efficacy

Pre-treatment thyroid stimulating hormone (TSH) for patient 1 and 2 was 0.09mIU/L and 6.95mIU/L respectively (normal range 0.40–3.50mIU/L). Patient 1 received two doses of thyrotropin alfa (0.9 mg) with TSH response of > 500mIU/L at time of treatment (target TSH > 30mIU/L). Pre-treatment regimen was adjusted in patient 2 to a single dose of thyrotropin alfa, eliciting TSH response of 162.80mIU/L at time of treatment.

### Treatment

Initial nuclear medicine whole body scan at 4-hours post-^131^I administration for patient 1 demonstrated pooling of radioactive material in the stomach, later dispersing on scans at 20- and 27-hours. This was postulated to be secondary to the presence of phosphate binders which were withheld for patient 2. Nuclear medicine whole body scan at Day 3 showed iodine-avid activity in the thyroid bed of both patients, with no activity seen elsewhere. At Day 3, percentage uptake in the thyroid bed of the residual retained activity was estimated to be < 1% for patient 1 and approximately 10% for patient 2.

**Fig. 3:**
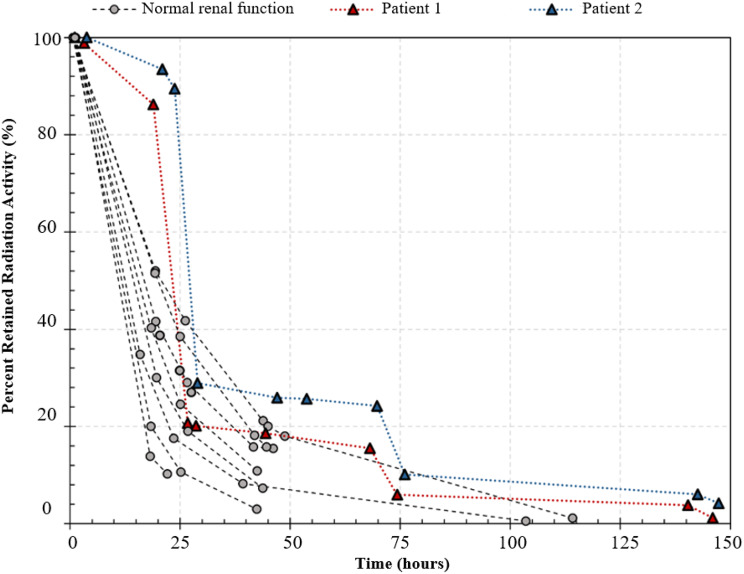
Retained radioactivity (%) over time. Haemodialysis sessions occurred at 24-, 72- and 144-hours post-^131^I administration at 0-hours

Retained radioactivity (%) as estimated from dose-rate meter measurements at 1 m distance over Day 0 to 6 is shown in Fig. 3. The first sessions of HD resulted in a reduction in radioactivity of 76% and 67% in patient 1 and 2 respectively. Interdialytic reduction in radioactivity (between HD sessions 1 and 2) was low at 5.2% and 4.7% for patient 1 and 2 respectively.

### Radiation dosimetry

Total radiation dose to blood was estimated to be < 0.3 Gy for both patients. Sixty percent and 47% of the radiation dose to blood was delivered in the time between ^131^I administration and first HD, for patient 1 and patient 2 respectively.

### Treatment outcomes

**Fig. 4:**
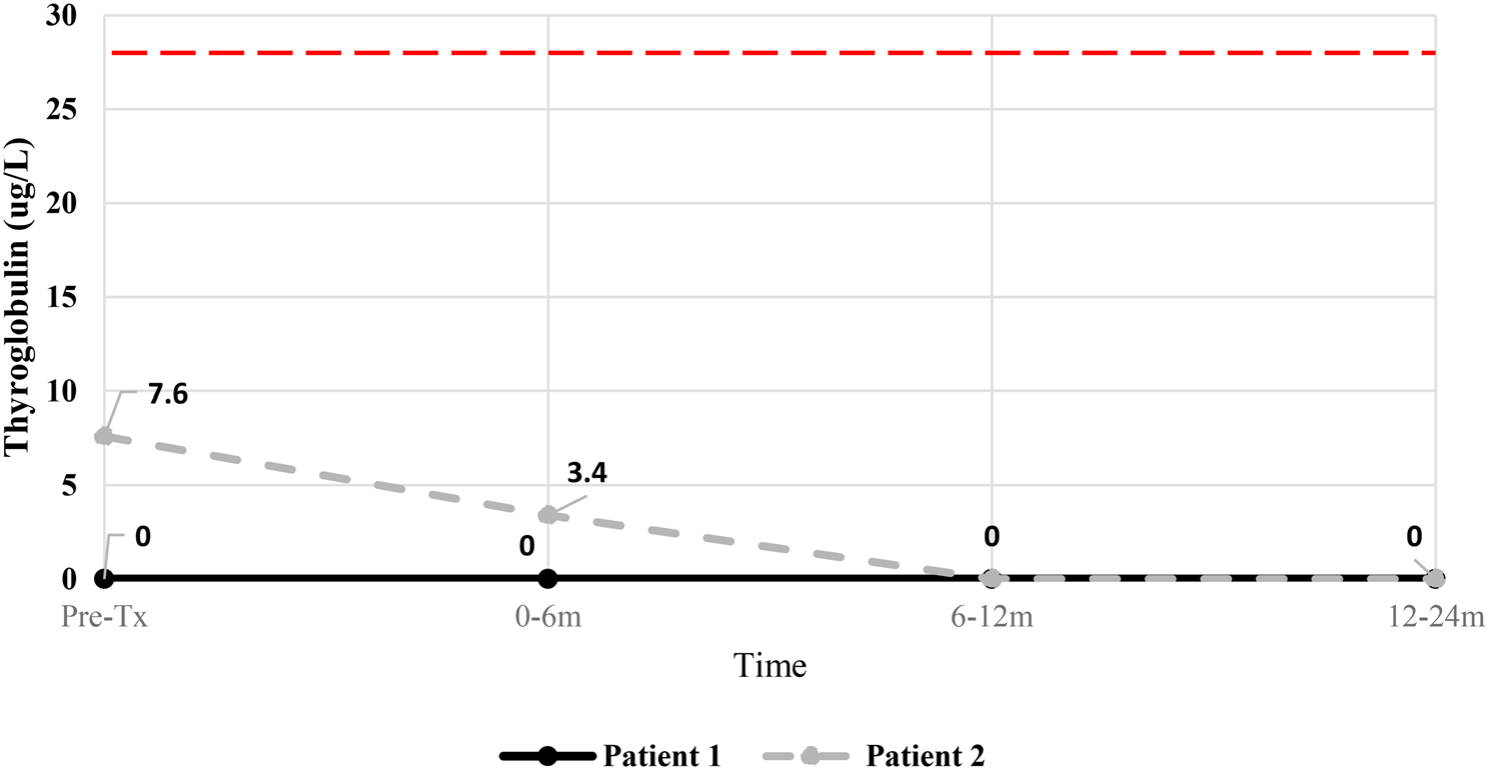
Thyroglobulin levels for patient 1 and 2 pre-^131^I treatment (post-thyroidectomy), at 0–6 months, 6–12 months and 12–24 months. Thyroglobulin upper normal limit (28.0ug/L) shown by red dotted line

Patient 1 had undetectable pre-treatment levels of thyroglobulin (normal range 0–28ug/L). Following ^131^I, thyroglobulin remained undetectable to 24-months post-treatment. Patient 2 had pre-treatment thyroglobulin of 7.6ug/L, declining to 3.4ug/L at 0-6-months post-treatment and became undetectable by 12–24-months. Thyroglobulin trends are shown in Fig. 4. Pre-^131^I -anti-thyroglobulin antibody level for patient 1 was 51.9IU/mL, declining to 22IU/mL at 6–12 months and 13.4IU/mL at 12–24 months (normal range < 4IU/mL) post-treatment. Anti-thyroglobulin antibody in patient 2 was undetectable pre- and post-^131^I.

### Nursing staff exposure

**Fig. 5:**
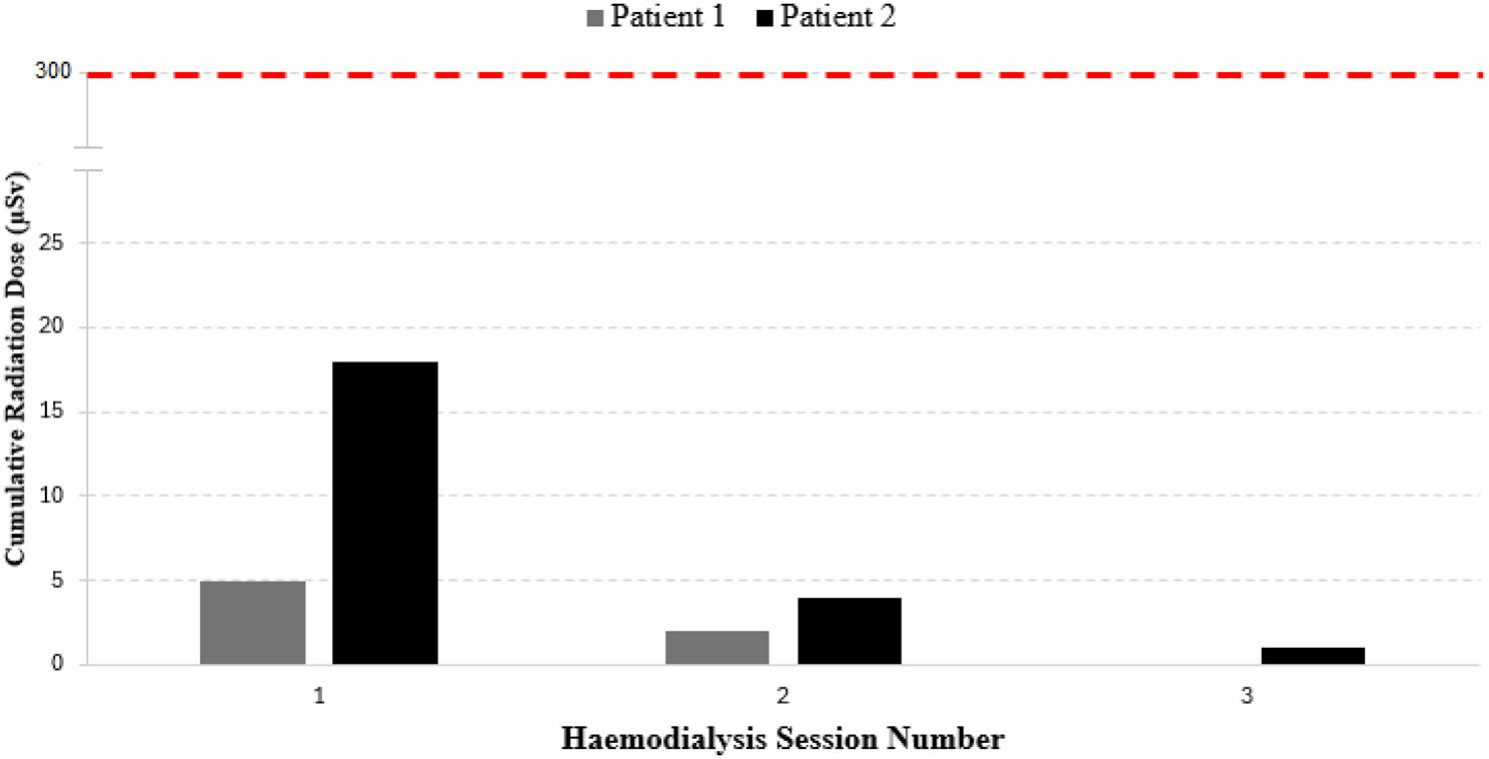
Cumulative radiation exposure to nursing staff across three haemodialysis sessions. Local constraints for safe radiation exposure threshold for the general public shown by red dotted line at 300μSv

No radioactive contamination was detectable on nursing staff PPE. Nursing staff exposure to radiation based on personal dosimeter readings is shown in Fig. 5. Cumulative nursing radiation exposure across 3 sessions of HD was 7μSv and 23μSv for patient 1 and 2 respectively, well within the local dose constraint of 300μSv per year for the general public. Actual radiation exposure to individual nurses was even lower as dialysis nurses were rotated at each session. Cumulative radiation exposure for nursing staff assigned to patient 1 was notably lower than staff assigned to patient 2.

### Equipment and radioactive waste

No radioactive contamination was detected on the HD machine. For each patient, a 12 L sharps bin containing HD machine disposables and needles used was stored as mixed biological-radioactive waste. A 5 L biological waste bin was stored containing stoma bags for patient 2.

## Discussion

The timing of first HD and the interval to subsequent HD after ^131^I administration is critical in optimizing treatment efficacy and minimizing risk of bone marrow toxicity. Previous studies have utilized a range of intervals to first HD (from 15-hours to 2 days) and subsequent HD (from 12-hours to 45-hours) [4] with variations in these regimens decided by the administered activity of ^131^I, readings of dose-rates from the patient, individual patient dialysis requirements and resource availability. Our decision for first HD at 24-hours and subsequent intervals of HD reflects our prioritization of minimizing treatment toxicity and meeting dialysis requirements, as both patients were in low to intermediate risk categories.

The timing to first HD is of particular importance as it determines the majority of radiation dose to bone marrow. For patient 1 and 2, 60% and 47% respectively, of the total radiation dose to bone marrow was delivered between ^131^I administration and the first HD, measured at 0.15 Gy and 0.1 Gy respectively. As the majority of ^131^I clearance is through HD, it follows that increasing the interval between ^131^I administration and the first HD session will significantly increase radiation delivered to bone marrow. In our patients, if first HD was scheduled at 48-hours post-administration, the calculated radiation dose delivered to bone marrow would increase to 0.3 Gy and 0.2 Gy for patients 1 and 2 respectively, but still remaining well within the accepted maximum tolerated dose to blood of 2.0 Gy [10], with more recent evidence indicating that even absorbed doses up to 3.0 Gy may be safe [11].

Additional factors affecting HD clearance of ^131^I were considered. Firstly, the clearance of ^131^I during and between dialysis depends primarily on ^131^I availability in blood. As the amount of ^131^I circulating in blood is inversely dependent on residual thyroid tissue, patients with greater thyroid remnants will have reduced inter- and intra-dialytic clearance. Patient 2 had greater ^131^I uptake in the thyroid bed than patient 1, explaining the lower inter- and intra-dialysis clearance observed. Another consideration is HD prescription. Both patients in the study utilized high surface area dialyzers at standard blood flow rate (300 mL/min) and HD duration. It is reassuring that the estimated radiation dose to bone marrow for both patients were well within safety thresholds, demonstrating a buffer for those with a smaller HD prescription and thus less clearance capacity.

Performing HD sessions at 24-, 72- and 144-hours (Days 1,3 and 6) post-^131^I produced a retained percentage radioactivity profile (i.e., overall clearance rate) comparable to patients with normal renal function (Fig. 3). This HD regimen also mirrors the schedule for most patients who undergo three times per week intermittent HD, minimizing the risk of needing emergent dialysis (e.g., for fluid overload, hyperkalaemia) whilst operating within local resource constraints. As expected, clearance of ^131^I between HD sessions was mainly due to physical decay of the radionuclide. Patient 2, who had a creatinine clearance of 6 mL/min, did not show any greater clearance of radioactivity than Patient 1, who was anephric, indicating that the typical residual renal function of a chronic HD patient is not able to significantly contribute to inter-dialytic ^131^I clearance.

The dosage of ^131^I is another uncertain factor, with conflicting evidence on whether to reduce, maintain or increase the standard dose of ^131^I given the prolonged half-life and reduced clearance of ^131^I in ESKD. Vermandel et al., in a case series of six patients, found a 30% reduction to standard ^131^I dose achieved a balance of treatment efficacy with bone marrow toxicity [8]. Holst et al., reached similar conclusions using mathematical modelling [12]. Other studies, conversely, have recommended equivalent dosing or increased dosing of ^131^I given higher clearance rates on HD and using individualized dosimetry to guide HD scheduling [13–15]. Although our data is limited to only two patients, assuming that HD clearance of ^131^I is independent of administered dose, it suggests that the administration of higher levels of radioactivity (up to 4GBq for patients with higher risk cancers) can theoretically be given to ESKD patients when the first HD session is scheduled at 24-hours post-administration. The safety margins for bone marrow toxicity in this instance however, becomes narrower with less flexibility in HD prescription, and ongoing radiation exposure measurements during treatment are crucial.

Regarding treatment efficacy, although the absence of disease recurrence in 12–24 months post-treatment in two low to intermediate risk patients may not be sufficient to conclude the efficacy of treatment, it is reassuring that no evidence of abnormality on subsequent follow-up ultrasound was detected and persistently undetectable thyroglobulin levels were observed in both patients.

Staff safety is another important dimension of administering ^131^I. Patients with normal renal function undergoing ^131^I radioablation treatment in hospital are usually isolated with minimal staff contact during their admission. However, patients requiring dialysis represent a deviation from routine practice, as dialysis nursing staff are required to be in close proximity to patients during sessions, when there would be increased risk of personal contamination and exposure to γ-radiation from ^131^I. These risks can be significantly mitigated with appropriate distancing, sensible positioning whilst preparing for HD and remote monitoring during HD. In our study, we achieved an overall cumulative radiation exposure to dialysis nursing staff that was very low, consistent with other studies [3]. Furthermore, as the closest and most prolonged patient contact occurs during fistula cannulation, if the patient can be safely trained to self-cannulate, we showed that radiation exposure can be reduced even further, seen in the notably lower cumulative staff radiation exposure for patient 1 compared to patient 2 (Fig. 5).

Finally, our experiences with patient 1 during the study led to additional protocol modifications which were applied to patient 2. One such modification was the suspension of phosphate binders prior to treatment as we suspect this may have resulted in the aggregation of ^131^I in the gastrointestinal tract seen on the 4-hour scan for patient 1. There is no literature studying the affinity of phosphate binders with ^131^I, but given it is a non-critical medication, withholding all phosphate binders prior to therapy is a reasonable approach. We also found the reduction in thyrotropin alfa to a single dose from the standard of two, produced a sufficient response in TSH to proceed with treatment in keeping with EANM guidance [8].

## Conclusions

The use of radioiodine in patients with ESKD on HD presents logistical hurdles and additional considerations for patient and staff safety. Our study supports the consensus that radioiodine can be safely administered to patients with low-risk thyroid cancer on HD if performed with additional precautions. The existing literature conveys a wide variation in protocols and there is not yet a standardization of approach to therapy in this population. Due to multiple patient variables and differences in resourcing across centers, it is not possible or practical to set out definitive parameters, but rather guidelines and practice points based on multi-center experiences will be invaluable for centers that encounter this scenario for the first time. Our modified protocol outlines one approach to radioiodine in patients on chronic HD to add to published experience and contribute to the future development of standardized guidelines.

## Data Availability

The datasets analyzed during the current study are not publicly available due to protect patient privacy, but are available from the corresponding author on reasonable request.

## Abbreviations

ATA: American Thyroid Association
CCTV: Closed circuit television
DTC: Differentiated thyroid cancer
EANM: European Association of Nuclear Medicine
ESKD: End-stage kidney disease
GBq: Giga-becquerel
HD: Haemodialysis
HREC: Human Research Ethics Committee
^131I^: Radioiodine therapy
NSW: New South Wales
PPE: Personal protective equipment
TSH: Thyroid stimulating hormone
